# Human tactile detection of within- and inter-finger spatiotemporal phase shifts of low-frequency vibrations

**DOI:** 10.1038/s41598-018-22774-z

**Published:** 2018-03-09

**Authors:** Scinob Kuroki, Shin’ya Nishida

**Affiliations:** NTT Communication Science Laboratories, Nippon Telegraph and Telephone Corporation, Kanagawa, Japan

## Abstract

When we touch an object, the skin copies its surface shape/texture, and this deformation pattern shifts according to the objects movement. This shift pattern directly encodes spatio-temporal “motion” information of the event, and has been detected in other modalities (e.g., inter-aural time differences for audition and first-order motion for vision). Since previous studies suggested that mechanoreceptor-afferent channels with small receptive field and slow temporal characteristics contribute to tactile motion perception, we tried to tap the spatio-temporal processor using low-frequency sine-waves as primitive probes in our previous study. However, we found that asynchrony of sine-wave pair presented on adjacent fingers was difficult to detect. Here, to take advantage of the small receptive field, we investigated within-finger motion and found above threshold performance when observers touched localized sine-wave stimuli with one finger. Though observers could not perceptually discriminate rightward from leftward motion, the adaptation occurred in a direction-sensitive way: the motion/asynchronous detection was impaired by adapting to asynchronous stimuli moving in the same direction. These findings are consistent with a possibility that human can directly encode short-range spatio-temporal patterns of skin deformation by using phase-shifted low-frequency components, in addition to detecting short- and long-range motion using energy shifts of high-frequency components.

## Introduction

**Figure 1:**
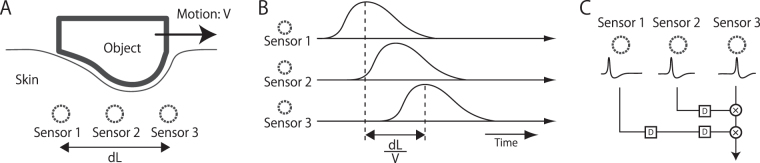
Conceptual sketch of spatio-temporal shift induced by input signal. (**A**) Skin deformation with a moving object. (**B**) Skin deformation at each sensor location. (**C**) Possible mechanism for encoding spatio-temporal shift between sensors. ‘D’ denotes delay. See text for further details.

Tactile signals are sensed by mechanoreceptors distributed over the elastic surface of the body, i.e., the skin. When the skin contacts an object, it is spatially deformed. As the skin or the body moves relative to the object, this deformation pattern is spatially shifted (Fig. 1A). This shift is the source of the brain’s ability to know the location changes or movements of an object on the skin (Fig. 1B). In other modalities, this type of spatiotemporal input pattern (e.g., inter-aural time differences in audition; motion in vision) is considered to be detected by coincidence detectors with delay lines^1,2^ or spatiotemporal energy detectors^3^. Similar mechanisms (Fig. 1C) are suggested to underlie tactile motion detection^4,5^.

In touch, there are two groups of sensory channels with different spatio-temporal characteristics^6–12^: One group comprises the Pacinian corpuscles (PC channel), which is sparsely distributed in a deep layer of the skin and sensitive to high-frequency vibrations. The other comprises the non-Pacinian (non-PC) channels, including Meissner corpuscles (RA channel) and Merkell cells (SA channel), which are densely distributed in a shallow layer of the skin and sensitive to low-frequency vibrations. While the PC channel has large receptive fields (e.g., covering the whole palm with transient stimuli), the non-PC channels have small receptive fields (2–3 mm on the finger skin). Considering these receptive field characteristics, the non-PC channels appear to be more suited than the PC channel for detecting spatiotemporal shift patterns on the skin surface. Indeed, many studies have reported behavioural and neuronal evidence indicating that the non-PC channels are related to tactile motion/direction detection^13–15^.

**Figure 2:**
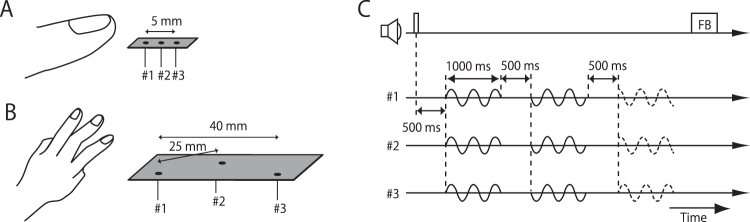
Stimulus arrangements and time sequence. (**A,B**) Participants placed their left index, middle, and/or ring fingers on the stimulators and boards in three-pin conditions. In two-pin conditions, pin #2 was removed and only pin #1 and #3 were used. (**C**) The waveform was fixed except for the phase. Two of the three pairs were synchronous (solid) and one of the first or third pair was asynchronous (dashed). Participants were asked to report whether the odd one was the first or third pair.

If tactile motion is computed by the motion detectors (Fig. 1C) using the input signals from the non-PC channels, we expect that the stimulus in Fig. 2C will be an effective inducer of tactile motion sensation. The stimulus consists of two or more sine-wave vibrations presented at multiple skin locations, with temporal phase shifts among them. The asynchronous stimulus produces a primitive motion input, i.e., a spatial shift of the temporal features (e.g., peaks) of the vibrations. The frequency of vibration is within the low-frequency range preferred by the non-PC channels (<40 Hz), where the information about the input waveform is well stored in the pattern of neural firings^12,16–22^. The phase difference among the low-frequency sine waves matches the behavioural effective range of tactile temporal judgments (20–100 ms)^23–27^.

Recently, we used this type of stimulus to psychophysically examine the human ability to detect spatiotemporal shifts of tactile inputs, and tactile movements, between adjacent fingers^28^. We presented a pair of low-frequency sine-wave vibrations to the index and middle fingers of the same hand of the participant, who was asked to discriminate an asynchronous (phase-shifted) pair from synchronous ones. The results showed that the asynchrony detection was nearly impossible (except when the vibration was so slow that the participants could use a cognitive tracking strategy). Since spatiotemporal asynchrony detection is a prerequisite to motion detection, failure in asynchrony detection implies the inability of the tactile system to detect motion (while success in asynchrony detection does not necessarily imply the ability to detect motion). In contrast, the participants’ performance was much improved when the sine-wave modulation was replaced with modulations with high-frequency components, such as a repetitive impulse sequence and an amplitude modulation (AM) of high-frequency vibrations. The results suggest that the PC channel, rather than the non-PC ones, contribute to spatiotemporal processing of tactile signals under the conditions tested in that study.

In this study, we tried to gain insight into the motion detection mechanism that takes low-frequency inputs (Fig. 1C) by presenting the sine-wave motion stimuli on a single finger. Compared to the inter-finger motion tested in Kuroki *et al*.^28^, within finger motion is more common in daily life. In addition, we conjecture that the tactile motion system may have a long-range motion mechanism and a short-range motion mechanism^29^ (see e.g.,^30^ for a similar idea in vision), and that the non-PC channels with small receptive fields may feed inputs to the short-rage (within-finger) motion mechanisms but not to the long-range ones.

We found that the performance of detecting asynchronous sine-wave stimuli (potentially containing a motion cue) from synchronous stimuli (containing no motion cue) was significantly improved when the stimuli were presented on a single finger (experiment 1A), but the performance was not as good as that with high-frequency components (impulse sequences and AM wave stimuli, experiment 1B). We could not find evidence of direction coding in a perceptual direction discrimination task (experiment 2); however, we found a direction-selective adaptation effect (experiment 3). These findings are consistent with the existence of short-range tactile motion detectors based on the non-PC channels with a small receptive field.

## Results

### Experiment 1A

The first experiment measured the threshold for discriminating an asynchronous stimulus from synchronous stimuli presented on the finger(s) and examined effects of the number of stimulated fingers and stimulators. Participants touched horizontally aligned pins of stimulators with their index, middle, and/or ring finger(s) of left hand (Fig. 2A,B). The presented stimuli were sine waves, and the phase difference between the rightmost pin and leftmost pin of asynchronous stimuli was ±90 degrees. To estimate synchrony/asynchrony discrimination sensitivity, we used odd-ball detection with two-alternative forced choice (2AFC). Participants were presented three pairs of stimuli (Fig. 2C) and asked to report one odd-ball stimulus (asynchronous “A”, which had a phase difference of ±90 degrees) from the other two stimuli (synchronous “S”). Here, the order of the three test stimuli was either A-S-S or S-S-A, and thus the chance level was proportion correct 0.5. This was a sensitive procedure to evaluate perceptual discrimination performance between A and S. We did not ask participants to identify the synchrony/asynchrony for a single stimulus presentation, since our preliminary observation suggested that some participants mistakenly identified S stimuli as A due to a response bias or a labelling error. The odd-ball task excludes the influences by such non-perceptual errors. Note also that, for each stimulus presentation, we randomized the initial phase of the stimulus so that the first or last pin position could not be a cue to discriminate A from S.

**Figure 3:**
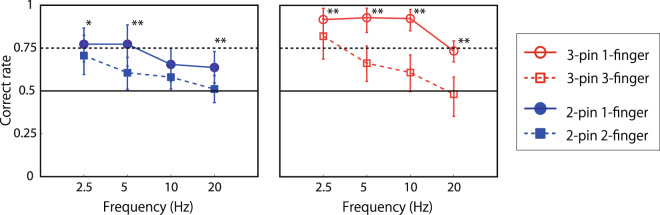
Results of asynchrony discrimination with sine waves (experiment 1A). Data points show averaged data of asynchronous discrimination performance across the ten participants with error bars representing 95% confidence intervals, which were calculated by the boot strapping method^58^. The vertical axis represents the ratio of asynchronous pairs detected correctly. The horizontal axis represents the frequency of presented stimuli. Asterisk denotes a significant difference between two conditions in the same graph in each frequency condition. **CI99% *CI95%. See detail in analysis section.

Figure 3 shows that the asynchrony discrimination performance (vertical axis) for all conditions decreased with an increase in frequency from 2.5 to 20 Hz (horizontal axis). First, we successfully replicated our previous result. The 2-pin 2-finger condition was identical to that in our previous study^28^, and the performance (filled blue squares) was low for 5- to 20-Hz stimuli. Though the performance was improved with 2.5-Hz stimuli, as in the previous study, this exceptional performance may reflect the contribution of the cognitive tracking mechanism (see discussion in^28^). When the stimuli were presented on the same finger (filled blue circles), the performance improved. When the number of stimuli was increased to three (right panel of Fig. 3), the performance improved, presumably because three pins produced asynchrony signals not only between pin #1 and #3, but also between #1 and #2, and between #2 and #3. Moreover, the performance for the one-finger condition (open red circles) far exceeded the 75% threshold and was clearly better than for the multiple-finger condition (open red squares). The performance in the 3-pin 1-finger condition dropped to 75% at 20 Hz, where the phase difference between the adjacent pins was 12.5 ms. This is close to the previously reported best performance of human temporal discrimination with single taps^23–27^.

### Experiment 1B

**Figure 4:**
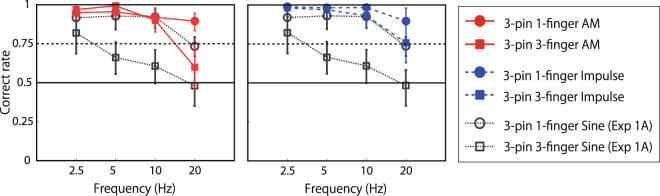
Results of asynchrony discrimination with AM waves and impulse sequences (experiment 1B).

We also measured the asynchrony detection performance with stimuli containing high-frequency components. The presented stimuli were AM waves (carrier frequency: 180 Hz) and impulse sequences. The phase difference was determined according to the interval between peak values (i.e., energy-based phase difference). Apparently, the performance was much higher than that with sine waves even with multiple-finger conditions (Fig. 4).

### Experiment 2

We found that asynchronous detection performance was above the 75% threshold when sine-wave stimuli were presented on a single finger pad. Though this is consistent with the existence of short-range tactile motion detectors sensitive to low temporal frequency inputs, success in asynchrony detection does not necessarily imply the ability to detect motion, since the asynchrony detection could be based on non-motion cues. One possible non-motion cue is produced by the spread of surface waves on elastic skin. The strain energy distribution (SED) on a finger pad with synchronous stimuli and asynchronous stimuli is likely to be different when stimuli are presented on one finger. Since there is no SED interference between stimuli when they are presented on separate fingers (multiple-finger condition), our result is consistent with this possibility. Another possible clue is a difference in lateral inhibition: stronger inhibition would occur in the synchronous condition, especially with the within-finger condition. Thus, participants might use the perceived intensity difference to find an odd “A” from the “Ss”. The critical test to tease apart the motion-based cue from the non-motion alternatives is to check direction sensitivity, which was done in the next two experiments.

The second experiment examined whether the participants could judge the direction of the phase shift of asynchronous sine-wave stimuli. The participants’ task was to discriminate an asynchronous stimulus with phase difference phi from an opposite asynchronous stimulus with phase difference –phi. The SEDs of the two asynchronous stimuli were mirror images, and expectedly hard to discriminate only from their spatial patterns. Furthermore, the inhibition strength is likely to be similar for asynchronous stimuli with ± phi, and thus the two asynchronous stimuli would be difficult to discriminate based on the inhibition magnitude cue. If, as shown in Fig. 1C, direction-sensitive motion detectors are involved, we expected the two asynchronous stimuli to be discriminable.

The presented stimuli were sine waves, and the phase difference was ±90 degrees. Participants were asked to report one odd-ball stimulus (one rightward shifted stimulus from two leftward shifted stimuli or vice versa). Since the initial phase of the stimulus was randomised, even when the rightmost pin protruded first, the stimulus was not necessarily a leftward one.

**Figure 5:**
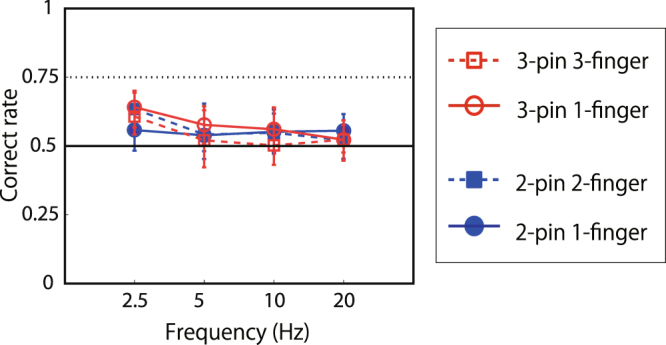
Results of direction discrimination with sine waves (experiment 2).

The result showed that in most cases, participants could not discriminate the two asynchronous directions (Fig. 5) even though the magnitude of asynchrony was above the detectable level in some conditions (Fig. 3). The results do not reject the possibility that the asynchrony detection in experiment 1 was based on a non-motion cue.

### Experiment 3

In contrast to experiment 2, experiment 3 supported the existence of motion detectors driven by phase-shifted sine-wave vibrations. In this experiment, we tested a direction-selective adaptation effect wherein adapting to asynchronous stimuli in one direction reduces the detection sensitivity to the asynchronous stimuli that have the same direction more than it does that to the asynchronous stimuli in the non-adapted direction. It has been shown that adaptation is an effective psychophysical technique for probing early sensory processing even when the outcome of that processing is not reflected in final perception^31–33^. Likewise, if there are direction-sensitive motion detectors in the early tactile processing, the adaptation effect may occur in a direction-sensitive way even when the final perception is not direction sensitive. A direction-selective adaptation effect itself has been observed in tactile modality with energy-based phase difference of impulse sequence and AM stimuli^34–36^. In the last experiment, we investigated direction-selectivity of motion detectors encoding spatio-temporal patterns of skin deformation by testing whether adaptation to asynchronous low-frequency sine wave stimuli affects asynchrony detection of succeeding stimuli.

**Figure 6:**
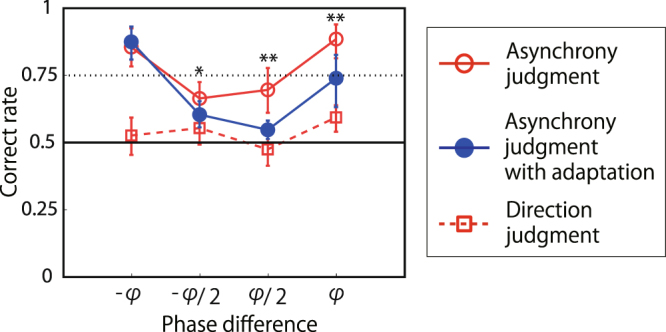
Results of asynchrony discrimination with/without adaptation (experiment 3). Red open circles represent asynchrony discrimination with the same procedure as in experiment 1 with 5 Hz stimuli with 90 degrees (phi) or 45 degrees (phi/2) asynchronous. Blue closed circles represent the same discrimination after 10-s adaptation to asynchronous stimuli of 5 Hz with phase difference phi. Red open squares represent direction discrimination with the same procedure as in experiment 2. Asterisk denotes a significant difference between with- and without-adaptation conditions in each asynchronous condition. **CI99% *CI95%.

The performance of sine-wave asynchrony detection was measured in the same manner as in experiment 1. The pin-finger combination was fixed to the 3-pin 1-finger condition. The presented stimuli were sine waves, and the frequency was fixed at 5 Hz. In the rightward/leftward adaptation condition, asynchronous stimuli with a phase difference of 90/−90 degrees were presented for 10 s. After that, three stimuli pairs were presented with in the order of either A-S-S or S-S-A, where the phase difference of the asynchronous pair was one of {−90, −45, 45, 90} degrees. Participants were asked to report one odd-ball stimulus (asynchronous “A” from the two “Ss”). The results for the with-adaptation condition were plotted according to the phase to which the participants had adapted: the data for the leftward (−90 degrees) adaptation condition were flipped and merged with those for the rightward (90 degrees) adaptation condition. Positive values on the horizontal axis mean the same direction as the adapted direction. Without adaptation (red open circles with solid lines in Fig. 6), the performance of the asynchrony detection of a test stimuli changed in accordance with its phase difference regardless of its direction: a small phase difference resulted in poor performance. When the performance was measured after adaptation (blue filled circles with solid lines), the detection performance depended not only on the absolute phase difference but also on the phase to which the participants had adapted: asynchronous with the same direction φ to adaptation stimuli was difficult to detect in the sine-wave condition. In addition, CIs were not overlapped between the with- and without-adaptation conditions for the phase difference of φ/2 to which the participants had adapted. Note that with these test stimuli, participants had no clear idea of their direction (red squares).

## Discussion

To investigate whether the brain can directly encode phase shifts of low-frequency inputs, especially with within-finger short-range motion, we presented continuous sine-wave stimuli on finger(s) with a phase difference, and measured the performance of asynchrony detection and its direction (sign of relative phase) discrimination. Experiment 1 demonstrated that participants could discriminate asynchronous stimuli of three sine waves from synchronous ones when the stimuli were presented on a single finger. The performance degraded when stimuli were presented by fewer pins or presented on multiple fingers, while that with AM waves or impulse sequences remained high both with single- and multiple-finger conditions. It is conceivable but unlikely that the observed discriminability was the result of feature tracking with attentional mechanisms, since the revealed discriminable time/phase difference (around 10 Hz) was much higher than the main target range of attentional mechanisms (2–3 Hz)^37^. In experiment 2, we found that participants could not perceptually discriminate asynchronous stimuli from the other asynchronous stimuli in the opposite direction (in which the absolute value of relative phase was the same but the sign was inversed), even when the asynchrony of the stimuli itself was detectable. It is possible that participants solved experiment 1 by using not a motion cue but the temporal pattern difference of the SED and/or a perceived intensity difference caused by lateral inhibition. If true, an adaptation to asynchronous stimuli would impair the performance of asynchrony detection regardless of direction/sign of the stimuli. Nevertheless, in experiment 3, we found that the adaptation indeed occurred in a direction-sensitive way. Even though observers could not report/perceive the direction of adaptation nor the target stimuli, an aftereffect occurred. Taken together, these findings support the view that human observers have some mechanisms that directly encode short-range spatio-temporal patterns of skin deformation by using phase-shifted low-frequency vibrations as inputs.

Past physiological findings have suggested tactile motion detection mechanisms that appear to be related to the current finding. Some neurons in the primary somatosensory cortex and peripheral afferents show receptive fields comprising a central region with excitation, adjacent regions of synchronous inhibition, and/or lagged inhibition^4,15,38,39^, which make these neurons selective for particular motion signals. Note that most of these reports were about the non-PC channels, which were preliminarily identified according to the receptive field size and temporal response characteristics based on established criteria^12,13,40^. Indeed, the high sensitivity of the non-PC afferents/neurons to the motion stimuli has been reported^13,15,41,42^. On the other hand, that of the PC channel has been obscure, since the PC channel is known to be highly insensitive to the spatial properties of stimuli presented to their RFs^43^. In a psychophysical study, Gardner and Sklar^14^ presented moving bar stimuli using a pin array actuator that selectively activates only RA and PC afferents and found that motion and its direction are well discriminated by human participants. Since the PC channel showed too little spatial resolution, they concluded that motion perception is mainly based on the RA responses and showed that the amount of firing of RA populations stimulated by their stimuli was correlated with the behavioural performance of direction discrimination. To these previous findings, our study adds new behavioural evidence of a tactile motion detector based on the non-PC channels, which are sensitive to short-range motion and have a small receptive field.

Although the present results support the existence of a tactile motion detector with a small receptive field and slow temporal characteristics, some of our findings cannot be explained by only this mechanism. In the remainder of the paper, we first discuss why the mechanism, which is direction sensitive according to the adaptation effect, failed to induce direction perception. Then, we comment on the PC-based long-range/high frequency motion detectors.

### Lack of direction sensitivity?

As we observed a direction-selective adaptation effect, the asynchrony/motion detector tapped by our stimuli has direction sensitivity. However, we could not observe a clear ability to discriminate perceived direction even in the within-finger condition, and we do not have a full explanation for this negative finding. One might suspect that there were many procedural differences between the current and previous studies^24,26,44^ and this might cause the difference in the results. Since we tried to limit the frequency range, our stimuli were not brief pulse stimuli but continuous 1-s stimuli. In addition, since we used an odd-one-out procedure, participants had to wait for three stimuli to make one judgment, whereas the direction/temporal order was judged for each stimulus in the conventional procedure. We tried to check for possible effects of the long duration, repetitiveness, and limited frequency range of our stimuli, but none of them drastically improved the performance (see supplemental materials). Another possibility is labelling confusion. For behavioural spatiotemporal tasks (e.g., direction judgement: experiment 2), it is widely known that the task becomes difficult when the stimuli are presented close in space^23,24,26,45^. The reason behind this has been ascribed that direction judgments require a process for not only perceiving the stimuli as asynchronous, but also one for spatially labelling the order of the presented stimuli (right or left first)^46,47^. The latter process has been suggested to be cognitive and attention-related^48^, and it may cause labelling confusion with closely presented stimuli. However, we consider this hypothesis unlikely since we used an odd-one-out procedure, which is rather free from a spatial labelling process. Finally, recall that our stimuli were apparent motion in the sense that they were sparsely and vertically represented. In other words, our stimuli were not designed to induce a directional distribution of tangential force/displacement (i.e., shear force). Note that motion-related neural signal has been measured with apparent motion stimuli^42,49^. Nevertheless, it is highly unlikely in our daily life that motion on the fingertip occurs without shear force. Shear force can directly encode the direction of motion; therefore, the brain has a good reason to use this information. Indeed, most neurophysiological evidence of motion-related neural signal was obtained with real motion (e.g., tangential scanning/force stimuli)^50–53^. This difference may account for the low subjective direction perception.

### Low-frequency phase shift vs high-frequency energy shift

We consistently observed relatively better performance of asynchrony detection with AM waves and impulse sequences compared to that with sine waves. In our previous study^28^, we discussed two kinds of neural responses that can act as the information source of timing signal for asynchrony detection: the phase-locked response to the phase of relatively slow inputs and the response to the amplitude change of relatively fast inputs (i.e., the loci of stimulus energy concentration). Accordingly, the stimuli we used in the current study can be described as follows: the low-frequency sine wave contains a phase cue, the high-frequency limited AM wave contains an energy cue, and the impulse sequence contains both the phase and energy cues. Taken together with this, our results support the dominance of the high-frequency energy cue in asynchrony detection, especially in the multiple-finger condition. This is consistent with our previous study in which stimuli were presented on the two adjacent fingers^28^. Our conjecture here is that temporal relationships between long-range (across finger) tactile inputs are mainly judged based on the mechanism signalling the energy shifts of the envelope structure of high-frequency components, while those between short-range (within finger) inputs may be detected by the mechanism signalling the phase of the low-frequency components in addition to the energy-cue mechanism. This seems to be consistent with the difference in the size of the receptive field of responding channels: Long-range motion is mainly encoded by high-frequency components through the PC channel with a wide receptive field, while low-frequency components contribute to short-range motion detection through the non-PC channels with a small size receptive field. Note, however, that asynchrony between AM waves was well detected not only when stimuli were presented across fingers but also when they were presented within finger, suggesting a possible contribution of the PC channel with a large receptive field for short-range motion. Although we have little knowledge about the reason behind this at present, we speculate that position information and timing information may come from different channels and then be interpreted as motion. The PC channel is known for its relatively large receptive field, and the position/direction information of within-finger stimuli in the current study (5 mm apart) would be highly obscure if it had been encoded only with this channel. The non-PC channels are known for their small receptive field, and even a single pulse stimulus on this channel can induce localized perception. Thus, it seems reasonable that the non-PC channel information is used as the position source. Recent physiological studies have shown an interaction between cross-channel information at a very early level of the somatosensory cortex^39,54,55^ or even at the spinal level^56^. This kind of seemingly hyper-resolution spatial acuity depending on information integration across different sub-modalities has been reported before^57^. Though the hypothesis remains highly speculative at this moment, it is a promising direction of future study to understand how these different characteristic channels are integrated and effectively contribute to our spatio-temporal perception.

In summary, the results suggest that human observers can detect within-finger motion/phase differences using phase information of low-frequency inputs. Asynchrony of the phase difference (i.e., motion cue) was detected when stimuli were presented on a single finger. Though the reason behind this remains obscure, the direction of motion stimuli could not be detected even when the asynchrony of the stimuli was at a detectable level. Nevertheless, the adaptation procedure revealed that the motion detector tapped by our stimuli has direction sensitivity. Though further investigations are necessary, our results are mostly consistent with the idea that human observers have a motion mechanism with slow temporal characteristics, and this mechanism may specifically respond to within-finger short-range motion signal. In addition, our results also indicate that human observers can detect within- and across-finger motion using energy shifts of high-frequency inputs. There are multiple mechanisms for tactile motion detection.

## Methods

### Participants

One of the authors (SK) and 18 volunteers (15 females), aged from 21 to 44 years and all right-handed except one, participated in the experiments. Ten of them participated in each experiment, with partial overlaps of participants across the four experiments. They gave written informed consent before the start of the experiment. The volunteers had no specialized knowledge about psychophysical experiments and were unaware of the purpose of the experiments. Recruitment of participants and experimental procedures were approved by the NTT Communication Science Laboratory Research Ethics Committee and were conducted in accordance with the Declaration of Helsinki.

### Apparatus

We used the same voice coil actuators (EMIC, Kyoto, Japan, 511-A) as in our previous studies^35,37^. The maximum force was 15 N at 5 kHz. Piano wire “pins” were fixed as contactors on actuators, and they vertically deformed the skin through holes in metal boards as shown in Fig. 1. A rigid surround with a 1-mm gap between it and each pin prevented the spread of surface waves of the skin^59^. The diameters of the contactor and its hole were 1.0 and 3.0 mm, respectively.

There were four finger-pin conditions. In the 3-pin 1-finger condition and 2-pin 1-finger condition, the stimuli were delivered with three/two pins to the participants’ left index, middle, or ring fingers (Fig. 2A). Each finger was chosen with approximately equal probability. The distance between the leftmost (#1) and rightmost (#3) pins was 5 mm. In the 3-pin 3-finger condition, the stimuli were delivered with three pins to the participants’ left ring, middle, and index fingers. The distance between the leftmost (#1) and rightmost (#3) pins was 40 mm (Fig. 2B). In the 2-pin 2-finger condition, the stimuli were delivered with two pins (#1 and #3) to the participants’ left middle and index fingers with separation of 40 mm.

A participant sat at a table with the left arm resting on an armrest, with the left index, middle, and/or ring fingers placed on the actuators. The pins of the actuators always contacted the fingers throughout the experiment. The equipment was placed in front of the participant, a little to the left side. Participants made responses by clicking a mouse with their right hand. They performed experiments with their eyes open to maintain their arousal level, but an occluder prevented them from seeing the vibrations of the actuators. They wore earplugs and headphones, from which pink noise was presented continuously throughout the experiment to mask any noise sound made by the vibration of the actuators.

### Stimuli

We tested 2.5-, 5-, 10-, and 20-Hz sine waves, AM waves with a carrier frequency of 180 Hz, and impulse-sequence stimuli with an onset of 5 ms for each impulse. Stimulus intensity was controlled through output voltage, which corresponded to the force/intensity of each stimulus. The amplitude of each stimulus was 0.4, 0.4, 0.3, and 0.3 mm for the 2.5-, 5-, 10-, and 20-Hz sine waves, 0.05 mm for AM waves, and 0.06 mm for the impulse sequences with the finger of participant SK, measured with a laser displacement meter (KEYENCE LC-2440). These values were chosen to match subjective intensity across stimuli and determined based on a preliminary experiment. The duration of the stimulus was always 1000 ms. To prevent participants from judging phase differences from abrupt skin deformation during the onset and offset of stimuli, each stimulus had a 25-ms cosine ramp both at its onset and offset.

### Procedures

In the asynchrony detection experiments (experiments 11A and B), a beep sound was presented at the beginning of each trial, and three test stimuli, each of which lasted 1000 ms, were delivered to the participant at intervals of 500 ms (Fig. 2C). The presented vibrations were sine waves in experiment 11A and AM waves or impulse sequences in experiment 1B. Two of the three stimuli were “synchronous (S)” and the remaining one was “asynchronous (A)”. The order of the three vibration stimuli was either A-S-S or S-S-A, with equal probability. Participants made a binary response as to whether the first or the third stimulus was different from the other stimuli. After each response, feedback by an auditory tone let the participant know whether his/her response was correct or wrong. Note that we did not ask participants to identify the asynchronous stimulus since there was no guarantee that they could always perceive the physically asynchronous stimulus as “asynchronous.” Instead, we instructed them to perform an odd-one-out task, which should be possible if they could detect any difference between the asynchronous and synchronous stimuli. This odd-one-out procedure was also used in our previous study^28^.

Four finger-pin conditions (2/3-pin single-/multiple-finger condition) were tested in experiment 11A, and two conditions (3-pin single-/multiple-finger condition) were tested in experiment 1B. In 2-pin conditions, an asynchronous stimulus was presented with the phase difference of ±90 degrees. In 3-pin conditions, an asynchronous stimulus was presented with the phase difference of ±45 degrees on adjacent vibrations so that phase difference between the rightmost and leftmost vibration was ±90 degrees. The lags were presented to the rightmost and leftmost vibration with equal probability. With the relative phase fixed, the initial phase of the stimulus was randomly chosen for each stimulus. Thus, the onset timing (i.e., when the pins pushed on the skin) was different for each presentation. The finger-pin condition, the presented frequency, and waveform were fixed during a block (ten trials), so the “S” was always the same while phase difference (90 or −90 degrees) and presented order of “A” were changed, i.e., the difference between presented stimuli in the same block was only their initial phase and relative phase. This procedure was chosen to make participants focus and maximize their performance. Participants took a break longer than 10 min after two or three blocks. Twenty trials were performed for each condition. Each participant in experiment 11A performed 320 trials (20 trials x4 finger-pin conditions x4 frequencies x1 waveforms); each participant in experiment 1B performed 320 trials (20 trials x2 finger-pin conditions x4 frequencies x2 waveforms).

In the asynchrony discrimination experiment (experiment 2), the procedure was the same as in the asynchrony detection experiment (experiment 11A) with following exceptions. Two of the three stimuli were “rightward motion (R)” and the remaining one was “leftward motion (L)” or vice versa. The presented vibration was a sine wave, the initial phase of the stimulus was randomised, and the phase difference between the rightmost and leftmost vibrations was ±90 degrees. The order of the three vibration stimuli was R-L-L, R-R-L, L-R-R, or L-L-R with equal probability. As in the asynchrony detection experiment, participants made a binary response as to whether the first or the third stimulus was different from the other two. Each participant performed 320 trials (20 trials x4 finger-pin conditions x4 frequencies x1 waveforms).

In the asynchrony detection with adaptation experiment (experiment 3), two tasks were used according to the condition: synchrony-asynchrony discrimination with/without adaptation and direction discrimination without adaptation. The finger-pin condition was 3-pin 1-finger, the stimuli were sine waves, and the frequency was 5 Hz. No feedback tone was presented for this experiment. For the synchrony-asynchrony discrimination task with adaptation, a trial consisted of a 10-s repetitive presentation of an adapting asynchronous stimuli (phase difference between rightmost and leftmost pins were ±90 degrees) followed by a beep sound and a presentation of a test stimuli with a 500-ms interval between adaptation and test stimuli. The duration of adaptation stimuli was chosen in accordance with our previous tactile motion aftereffect studies^35,37^. The test stimuli were either A-S-S or S-S-A with the phase difference of asynchronous test stimuli “A” of {−90, −45, 45, 90} degrees. Participants made a binary response as to whether the first or the third stimulus was different from the others. The adaptation condition was no adaptation, rightward adaptation, or leftward adaptation during one block (five trials for each phase difference of test stimuli). Four blocks were performed for the no-adaptation condition. Two blocks were performed for the rightward and leftward adaptation conditions, and the results obtained from them were averaged over the relative phase difference between adaptation and test stimuli. To let participants make a good guess about the test stimuli, an adaptation block was always tested after a no-adaptation block, in which all variations of target “A” were presented within a short experimental time. An initial 20-s adaptation was made at the beginning of each adaptation block. Participants took a break longer than 10 min after each adaptation block to erase the aftereffect on the next block. To avoid fatigue of the finger pads, participants changed fingers for each block, the index finger or middle finger. Since the duration of one block was long in this experiment, the ring finger was not used to avoid finger cramps. Each participant performed 160 trials (20 trials x1 finger-pin condition x1 frequency x1 waveform x2 adaptation conditions x 4 phase differences).

We conducted the direction discrimination task with the same asynchronous test stimuli as in the adaptation condition. The procedure was the same as in asynchrony discrimination experiment (experiment 2) except that the presented test stimuli (L or R) were 5-Hz sine waves with a phase difference of {−90, −45, 45, 90} degrees. Participants made a binary response as to whether the first or the third stimulus was different from the others. Each participant performed 80 trials (20 trials x1 finger-pin condition x1 frequency x1 waveform x4 phase differences).

### Analysis

To quantitatively evaluate the response difference across conditions (difference in pin-finger combination in experiment 1, difference in adaptation in experiment 3), we calculated the index for each frequency condition (horizontal axis) using the boot strapping method. For each frequency condition, differences in proportion correct between two conditions were calculated for each participant, and they were used as seeds for the boot strapping method. An above zero value of the lower limit of CI means the difference between two conditions is significant. *In graphs indicates the lower limit of 95% CI was above zero; **denotes indicates the lower limit of 99% CI was above zero.

### Data availability

The datasets generated during and/or analysed during the current study are available from the corresponding author on reasonable request.

## Electronic supplementary material

Supplemental experiments
