# Supplementary material for: Human tactile detection of within- and inter-finger spatiotemporal phase shifts of low-frequency vibrations
Source: Sci Rep. 2018 Mar 9;8:4288. doi: 10.1038/s41598-018-22774-z (PMC5844903; doi:10.1038/s41598-018-22774-z)
Supplement: Supplementary file 1 — Supplemental experiments [file 41598_2018_22774_MOESM1_ESM.docx]

**Human tactile detection of within- and inter-finger spatiotemporal phase shift of low-frequency vibrations**

**Scinob Kuroki^1,*^ and Shin’ya Nishida^1^**

^1^NTT Communication Science Laboratories, NTT Corporation, Kanagawa, Japan

^*^ kuroki.shinobu@lab.ntt.co.jp; scinob@gmail.com

Supplemental materials

In the control experiments, we checked for effects of stimulus continuity, task difficulty, and fatigue on direction judgment in experiment 2

In the first control experiment (experiment S1), we used an impulse train with longer intervals instead of low-frequency sine waves. The reason behind this was to individually control motion impressions of the intended direction and unintended one. Theoretically, repetitive stimuli contain ambiguity of motion direction. Imagine there are three asynchronous sine wave stimuli presented with horizontal alignment on the skin surface. If the first wave of the leftmost stimulus precedes the middle one and the middle one precedes the rightmost one, the rightward motion component can be calculated. However, at the same time, an unintended leftward motion component emerges between the first wave of the rightmost stimulus and the second wave of the leftmost one (left panel of Fig. S1a). One can reduce binding of the unintended stimulus features by temporally separating them (e.g., by separating the first wave of the rightmost stimulus and the second wave of the leftmost one). Using this technique for the sine waves in our experiment is difficult because when the frequency became too low (e.g., to achieve 1000-ms interval/wavelength, 1 Hz is required) the perceptual threshold became too big. With impulse sequence stimuli, we can easily control the motion perception of the supposed direction by changing the temporal shift across trains (asynchrony of stimuli) and that of the unintended direction by changing the temporal interval within each train (pulse per second of each stimuli) (right panel of Fig. S1A).

Ten volunteers (five females), aged from 22 to 49 years and all right-handed, participated in the supplemental experiments. The apparatus and the procedure were identical to those in experiment 2 except for the following points. The pin-finger combination was fixed to the 3-Pin 1-finger condition. The presented stimuli were impulse-sequence stimuli with an onset of 5 ms for each impulse, and the asynchrony of stimuli and the interval between pulses were individually controlled.

The asynchrony (stimulus onset asynchrony: SOA) between leftmost and rightmost stimuli was {100, 50, 25, 12.5} ms, which was identical to {2.5, 5, 10 20} Hz sine waves that used in our main experiments. The interval between pulses was {1000 (this was equal to infinity, since the duration of the stimuli was 1000 ms), 600, 400, 200} ms, where the pulses per second were {1, 2, 3, 4}, respectively. Note that in the main experiment (experiment 2), since the asynchrony of stimuli was ±90 of the frequency of each stimuli, these parameters were entangled: when the stimulus frequency was 2.5/5/10/20 Hz, the asynchrony/SOA between stimuli was 100/50/25/12.5 ms, and the interval/wavelength between stimuli was 400/200/100/50 ms, respectively. Participants were asked to report one odd-ball stimulus (one rightward motion stimulus from two leftward motion stimuli or vice versa) (Fig. S1B).

The performance of direction discrimination with a long interval became better than that with a short interval (Fig. S1C). The horizontal and vertical axes represent the asynchrony/SOA between leftmost and rightmost stimuli, and the correct rate of the odd-one-out task. The blue close circles with the dotted line, green close circles with the solid line, red open circles with the dotted line, cyan open circles with the solid line represent the impulse intervals of 200, 400, 600, and 1000 ms, respectively. When the asynchrony/SOA was as long as 100 or 50 ms, the CIs of each condition were above chance level (0.5). In particular, when the interval was as long as 400, 600, or 1000 ms, the upper limits of CIs exceeded the threshold level (0.75). These results suggest not a high ability but some ability to discriminate the direction of slow motion with long intervals.


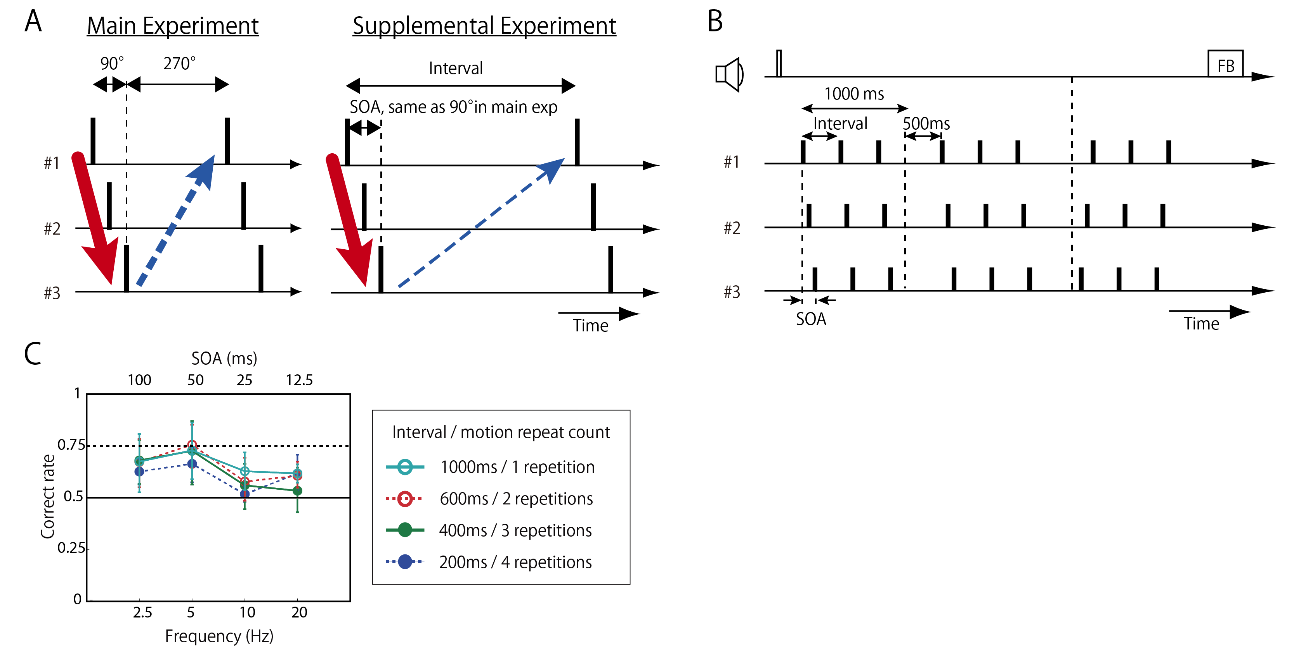
Figure S1.

1. Schematic diagram of stimuli used in experiments. Red solid arrows indicate supposed motion direction, where velocity-induced perceived intensity can be controlled by changing the SOA. Blue dotted arrows represent not supposed motion, where velocity cannot be individually controlled with supposed motion since it was always three times that of the supposed motion in the main experiments.
2. Time course of experiment S1. Participants were asked to report whether the odd one was the first or third pair.
3. The results of experiment S1. The vertical axis represents the ratio of different directional motion detected correctly. The horizontal axis represents the SOA (i.e., velocity of the expected motion; red solid arrows in panel A) of the asynchronous pair. Data points represent the average of the results for the ten participants with error bars representing 95% CIs.

In the second control experiment (experiment S2), we used the same stimuli as in experiment S1 (impulse train with longer interval) but with a different procedure: direct identification of motion direction. Originally, we used an odd-one-out procedure to reduce possible artifacts and labelling confusion. Let’s assume that if there would be no labelling artifact, the level of task difficulty in asynchrony detection procedure (A-S-S or S-S-A) is the same as that of direct identification (S or A). That is, if participants can differentiate stimulus A with t ms asynchronous from stimuli S with probability P(t), the correct rate of the odd-one-out and direct identification would be the same P(t). However, this equivalence does not work in direction discrimination tasks. Let’s assume that participants perceive stimuli R with t ms asynchronous as rightward with probability P(t), which distributes on a cumulative Gaussian like psychometric curve with P(t=0)=0.5, and the opposite is true, i.e., they perceive stimuli L with –t ms as leftward with same probability P(t) which distributes on the flipped psychometric curve. Here, the correct rate of direct identification would indeed be P(t), while that of the odd-one-out procedure would be 1-2*P(t)(1-P(t)), which is always below P(t) without labelling misses. For example, if the correct rate of the direct identification task P(t) is 0.75, that of the odd-one-out task will drop 0.625. Indeed, when we rechecked task performance with direction identification procedure instead of odd-one-out procedure, we found that it increased and was around threshold level. The apparatus and procedure were identical to those in experiment S1 except that only one asynchronous pair was presented and participants were asked to report whether it was a rightward motion stimulus (rightmost pin followed leftmost pin) or leftward motion one (Fig. S2A).

The performance of the direction identification task P(t) (Fig. S2B) was above that of the odd-one-out task (Fig. S1C), as expected. When the results from these two experiments are plotted as a scatter map, the score for experiment S1 (horizontal) was always below that for experiment S2 (vertical) (Fig. S2C, left panel). When P(t) was simulated by using the results of experiment S1, it matched the directly measured P(t) in experiment S2 (Fig. S2C, right panel).


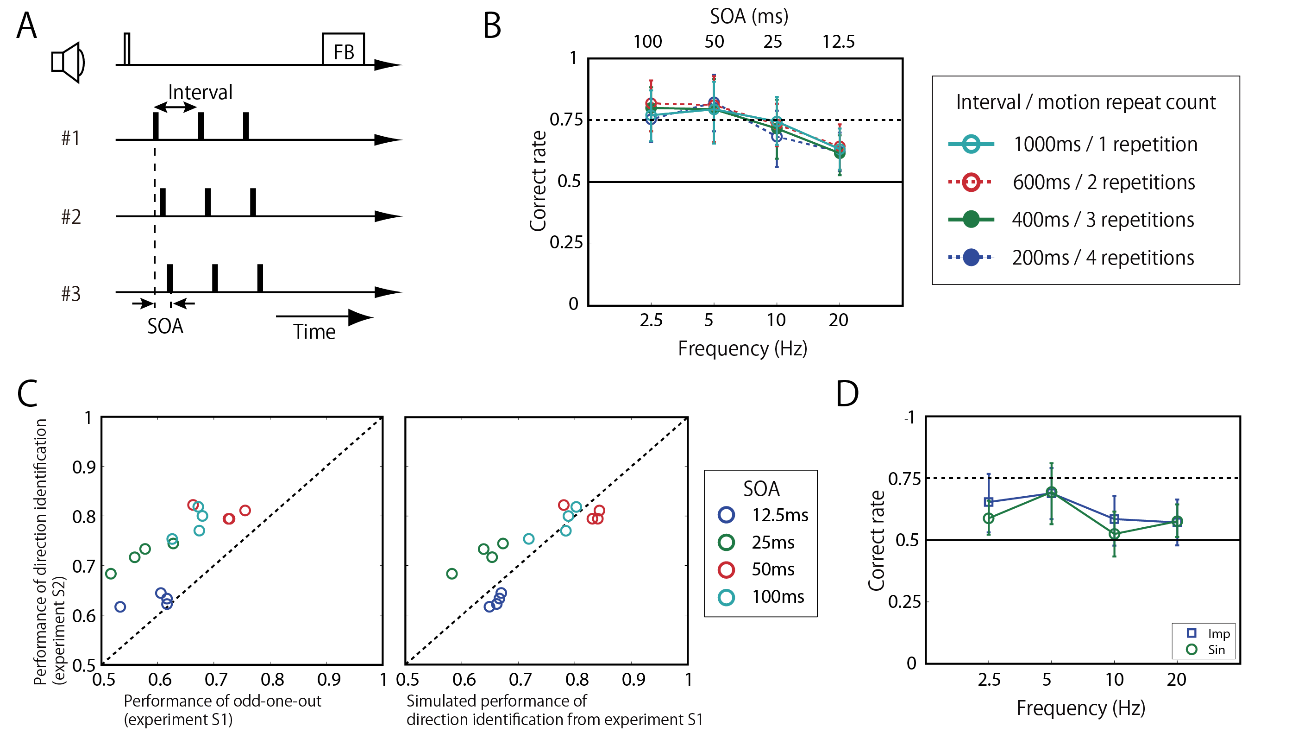


Figure S2.

1. Time course of experiment S2. Participants were asked to report whether the stimuli were rightward or leftward.
2. The results of experiment S2: Direction discrimination performance for impulse sequence with intervals.
3. Scatter map for comparing the performance of odd-one-out task with that of direct identification task. The plot in the left graph compares raw data. The horizontal axis represents data from experiment S1; the vertical axis represents data from experiment S2. The plot in the right graph shows simulated P(t) from experiment S1 and measured P(t) from experiment S2.
4. Direction discrimination performance with direction identification procedure for the sine waves and impulse sequence used in the main experiment.

Note that we checked task performance in direction identification procedure with continuous sine waves and impulse sequence stimuli with the same short intervals as those between the sine waves used in experiment 1. As shown in the Fig. S2D, the correct rate was slightly improved compared to that in the main experiment (Fig. 3), but Cis fell below the threshold level in most cases.

In the last control experiment (experiment S3), we measured the performance of direct identification of motion direction by presenting three pairs of stimuli. In addition to task difficulty, the simple effect of fatigue might be considered. Since the odd-one-out procedure requires exposure to three stimulus trains, while the direct identification procedure requires exposure to one, participants can get easily fatigued with the former. We checked this point by measuring direct identification performance by presenting odd-one-out stimuli. The apparatus and the procedure were identical to those in experiment S1, except that participants received auditory cues signaling which one was odd and were required to report the direction of odd one. As expected, the performance P(t) shown in Fig. S3B was slightly lower (fatigue effect observed) compared to that in experiment S2 (Fig. S3C).


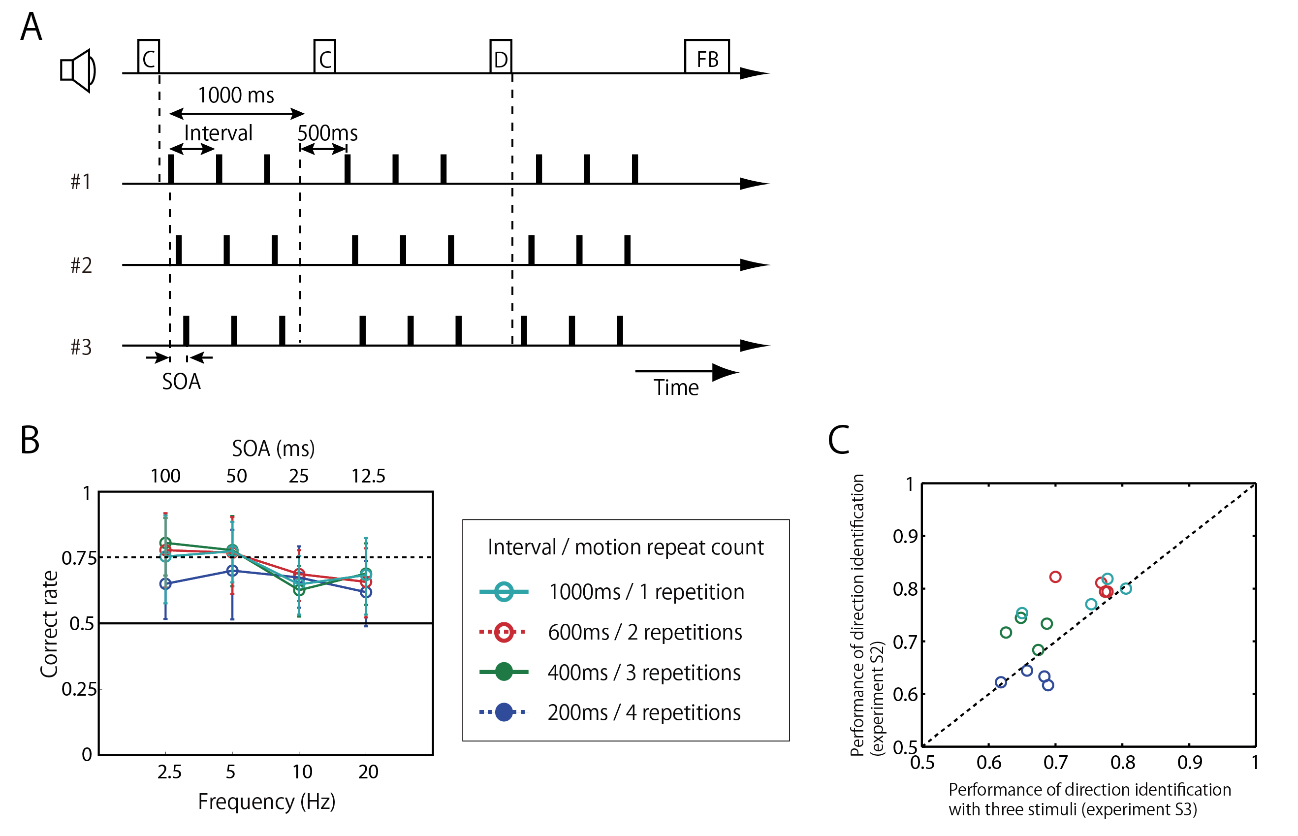


Figure S3.

1. Time course of experiment S3. Participants were asked to report whether the odd stimuli was rightward or leftward. They identified the odd one based on auditory cues (C note from two D notes or D note from two C notes). The trials in which participants failed to identify the odd one (i.e., participants missed auditory cues) were excluded.
2. The results of experiment S3: Direction discrimination performance for impulse sequence with auditory cues.
3. Scatter map for comparing performance of direction identify with one stimulus or three stimuli in which target stimuli were presented only once. The horizontal axis represents data from experiment S3; the vertical axis represents data from experiment S2.

In summary, we found reasonable performance of direction discrimination with impulse sequences when direction ambiguity was reduced by introducing intervals between directional signals. Note that introducing gaps between motion signals may facilitate attentional/feature tracking. In addition, removing effects of task difficulty and fatigue led to some improvement.

We cannot test low-frequency limited signals because a long enough interval cannot be introduced between amplitude peaks and direction ambiguity is unavoidable. According to the measured performance for impulse sequences, however, the performance for low-frequency stimuli would not reach perfect, though some improvement may or may not be observed. Further research is needed to investigate motion direction perception.
